# Effectiveness and Efficiency of Externally Bonded CFRP Sheets for Shear Strengthening of RC Beam-Column Joints

**DOI:** 10.3390/polym14071347

**Published:** 2022-03-26

**Authors:** Bu Wang, Xianhui Wu, Qi Liu, Yueyue Wu, Fei Huang, Linfeng Xu, Xing Wu, Yuanxin Deng

**Affiliations:** 1 School of Civil Engineering, Chang’an University, Xi’an 710064, China; wuxianhui2005@126.com (X.W.); 2019128031@chd.edu.cn (F.H.); spero7@163.com (L.X.); wuxing0614@163.com (X.W.); 2020128054@chd.edu.cn (Y.D.); 2 Dongying Transportation Development Group, Dongying 257091, China; liuqiytu@163.com; 3 Hebei TOP Architectural Design CO., Ltd., Shanxi Branch, Taiyuan 030000, China; wyy_0110@163.com

**Keywords:** carbon fiber-reinforced polymer, reinforced concrete beam-column joint, transverse beam, shear strengthening, nonlinear finite element analysis

## Abstract

To develop feasible carbon fiber reinforced polymer (CFRP) retrofit schemes for the shear strengthening of real three-dimensional reinforced concrete (RC) beam-column joints, a series of parameters in relation to the contributions of the CFRP sheets externally bonded to joint panels was numerically investigated in this study. The parameters include CFRP reinforcement ratio, CFRP layout, transverse beam-to-joint panel width ratio, transverse beam-to-joint panel height ratio, location of transverse beam, and number of transverse beams. Strengthening efficiency, a new dimensionless index, was introduced to evaluate the residual effect of a CFRP-strengthening system weakened by the presence of transverse beams in comparison with the increase in joint shear capacity in relation to the one-way counterpart. The results obtained from 44 nonlinear finite element models, which were calibrated against experimental observations, confirmed the effectiveness of the CFRP strengthening technique with regard to the relatively wide ranges of the parameters. The significant differences among the roles of the parameters were revealed, and the reasons behind the differences were analyzed. Furthermore, the shear mechanism of the CFRP-retrofitted joint panels was discussed with the proposed strut-and-tie model.

## 1. Introduction

The shear strengthening of reinforced concrete (RC) beam-column joints is a challenging and arduous task considering that large variations in both the geometry and the distribution of loads occur over relatively small volumes of joint panels. In comparison to the conventional strengthening materials, fiber-reinforced polymer (FRP) materials have several advantages, and these merits are widely discussed in the literature [1]. Externally bonded FRP systems are used to upgrade the various RC elements in the different loading scenarios [1,2,3]. From a practical standpoint, FRP materials may also have the potential to be used for the shear retrofit of RC joints.

For almost two decades since the late 1990s, extensive research has been conducted on FRP-strengthened joints. Investigations were performed on the roles of various parameters on the effectiveness of externally bonded FRPs [4,5,6,7,8,9,10,11,12,13,14,15,16,17,18,19,20,21,22,23], analytical models were built to predict the shear capacity of FRP-strengthened joints [9,20,24,25,26,27,28,29], and nonlinear finite element (FE) modeling approaches were developed for FRP-strengthened joints [30,31,32,33]. Nevertheless, most of the studies concentrated on two-dimensional (2D) joints, i.e., no transverse beams [34].

A few experimental attempts have been rendered for FRP-strengthened three-dimensional (3D) corner RC joints (i.e., each with a single transverse beam). Antonopoulos and Triantafillou [6] conducted a comprehensive experimental program on fifteen 2D exterior joints and three 3D corner joints that were strengthened in shear with various FRP schemes in relation to a series of parameters. The reverse cyclic loading tests of the joints demonstrated the effectiveness of the schemes. However, the results also indicated that the efficiency of the FRP sheets in the joints with a transverse beam was reduced in comparison with 2D specimens. Akguzel and Pampanin [10] tested six 2D exterior joints and four 3D corner joints under unidirectional and bidirectional reverse cyclic loading regimes, respectively. Three strengthening schemes were designed with glass fiber-reinforced polymer (GFRP) sheets and investigated to determine improvements in joint shear capacity. The test outcomes confirmed the potentially unconservative effects of neglecting actual multiaxial load demand. Del Vecchio et al. [15] presented an experimental investigation involving six 3D corner joints under reverse cyclic loading in as-built and FRP-strengthened configurations. Quadriaxial and uniaxial carbon fiber-reinforced polymer (CFRP) sheets were used in and out of the joint panel, respectively. The experimental results showed that the use of 0.004 as the designed maximum strain value of the FRP sheets applied to the joint panel seemed to be conservative. Mostofinejad and Hajrasouliha [18] experimentally verified the effectiveness of an L-shaped CFRP configuration with the grooving method in the shear retrofit of 3D corner joints. In the method, the grooves filled with epoxy resin were created at the concrete substrate to improve the bond behavior of CFRP sheets. Within a multi-objective retrofit scheme proposed by Pohoryles et al. [19], CFRP strands (i.e., rolled CFRP sheets were glued together using epoxy) were applied through the holes drilled through transverse beams to increase the shear capacity of 3D interior joint panels.

In most cases, transverse beams exist on both sides of a joint panel and the cross sections of transverse beams are much smaller than that of the beam (e.g., transverse beams designed as the link beams in the longitudinal direction of the gravity-load-designed RC frame structures). From a practical point of view, to develop reliable FRP retrofit schemes for the shear retrofit of real 3D RC joints, a comprehensive investigation is essential in determining the influence of a series of factors related to the presence of transverse beams on the effectiveness and efficiency of the CFRP sheets externally bonded in the different areas at joint panels, which depends mainly on the scenarios of transverse beams. In the current study, the nonlinear FE modeling approach was used to fill the knowledge gap. FE modeling is regarded as a cost-effective and reliable tool for simulating seismic responses of FRP-strengthened RC joints [30,31,32,33,35,36,37]. The parameters considered in this study were the following: (1) CFRP reinforcement ratio, (2) CFRP layout, (3) transverse beam-to-joint panel width ratio, (4) transverse beam-to-joint panel height ratio, (5) location of transverse beam, and (6) number of transverse beams.

## 2. Materials and Methods

### 2.1. Test Specimens and Loading

Two scaled-down (1/2) 2D exterior RC joints, namely, the original specimen (TJ-1) and the CFRP-strengthened specimen (TJ-3), were designed to simulate the nonductile detailing characteristics of pre-seismic code construction. The experimental results of both specimens were used to verify the accuracy of the modeling approach used in the current study.

**Figure 1:**
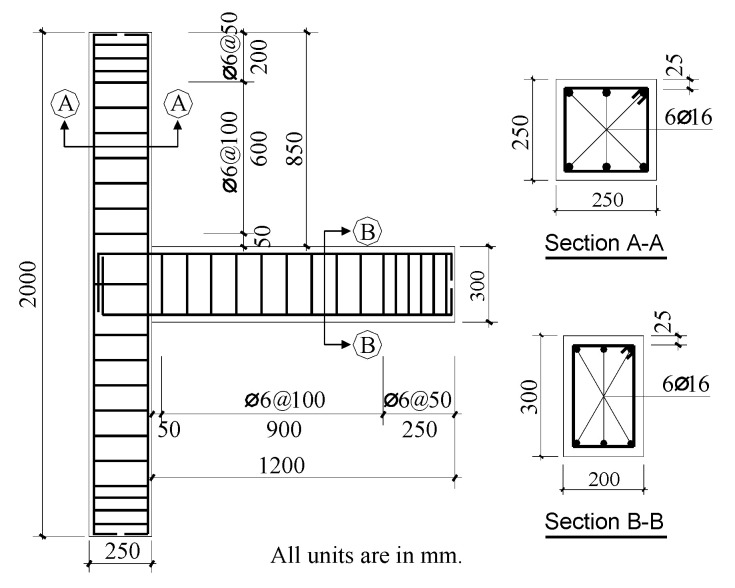
Geometry and reinforcement details (all dimensions are in mm; Ø and @ refer to the diameter of rebar and the stirrup spacing, respectively).

The geometry and reinforcement details of the specimens are shown in Figure 1 and were used to build the FE models in the current study. The strengthening scheme for Specimen TJ-3 is described in detail in Section 2.2. Considering the size effect due to the externally bonded CFRP-strengthening system, the relatively thin unidirectional carbon fiber sheets with a unit mass of 200 g/m^2^ (Toray Industries, Inc., Tokyo, Japan) instead of the prevailing ones with a unit mass of 300 g/m^2^ were used (i.e., the nominal thickness of the former is only 2/3 of the latter). The material properties associated with the specimens are presented in Section 2.4.

**Figure 2:**
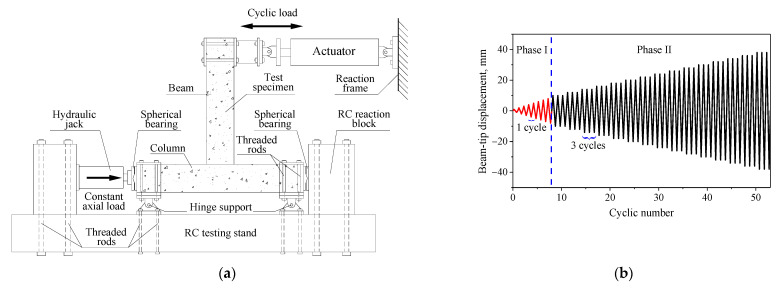
Test method. (**a**) Test setup; (**b**) Displacement history of cyclic loading.

The specimens were tested in a position rotated 90° from their existing position in a frame structure, as shown in Figure 2a. An axial load was applied at the upper column tip, which was exerted by a hydraulic load jack, and kept constant at the level of 200 kN during the test. After the full axial loading, a quasi-static cyclic load was applied at the beam tip (i.e., the outmost end of the beam) using a hydraulic actuator (MTS, Eden Prairie, MN, USA). The quasi-static cyclic load was applied at the beam tip in a displacement-controlled manner. A two-phase loading routine was used, as shown in the Figure 2b. In the first phase, the displacement of the beam tip in both push and pull directions began at 1 mm, and then increased to 2 mm, 3 mm, and so on, up to 8 mm. Each of the load steps had only one hysteretic loop. The second loading phase was then initiated with an increment of 2 mm. In this phase, every load step cycled three times. The failure of specimen was identified when the peak load in push or pull directions of the first loop in a load step decreased to 85% of the ultimate load recorded in the corresponding direction. The quasi-static loading rate was 0.2 mm/s.

The boundary conditions of Specimens TJ-1 and TJ-3 were also used for the FE models in the current study. However, a displacement-controlled monotonic loading was applied at the beam tip of each FE model to improve the modeling convergence behavior.

### 2.2. Strengthening Schemes and Specimen Matrix for FE Simulation

Three series of exterior joints, namely, Series D, R, and S, were considered in the current study. The reference specimen set, Series R, consisted of the one-way Specimens TJ-1 and TJ-3. The joint shear capacities of Specimens TJ-1 and TJ-3 were used as the lower and upper bounds of those of the CFRP-strengthened 3D joints, respectively. In the case of Series D, each side of the joint panel involved a transverse beam, and the two transverse beams were identical and existed at the same location. However, each of the joints in Series S had only one transverse beam. In accordance with the locations of transverse beams, the joints in Series D were further classified into three groups, namely, Groups DE, DM, and DI. The labels DE, DM, and DI represent the transverse beams in a joint aligned with the exterior edge, centerline, and inner edge of the joint panel, respectively. The side where the beam and the column intersect was called the inner edge, whereas the opposite side of the inner edge was referred to as the exterior edge. Similarly, Groups SE, SM, and SI existed in Series S.

**Figure 3:**
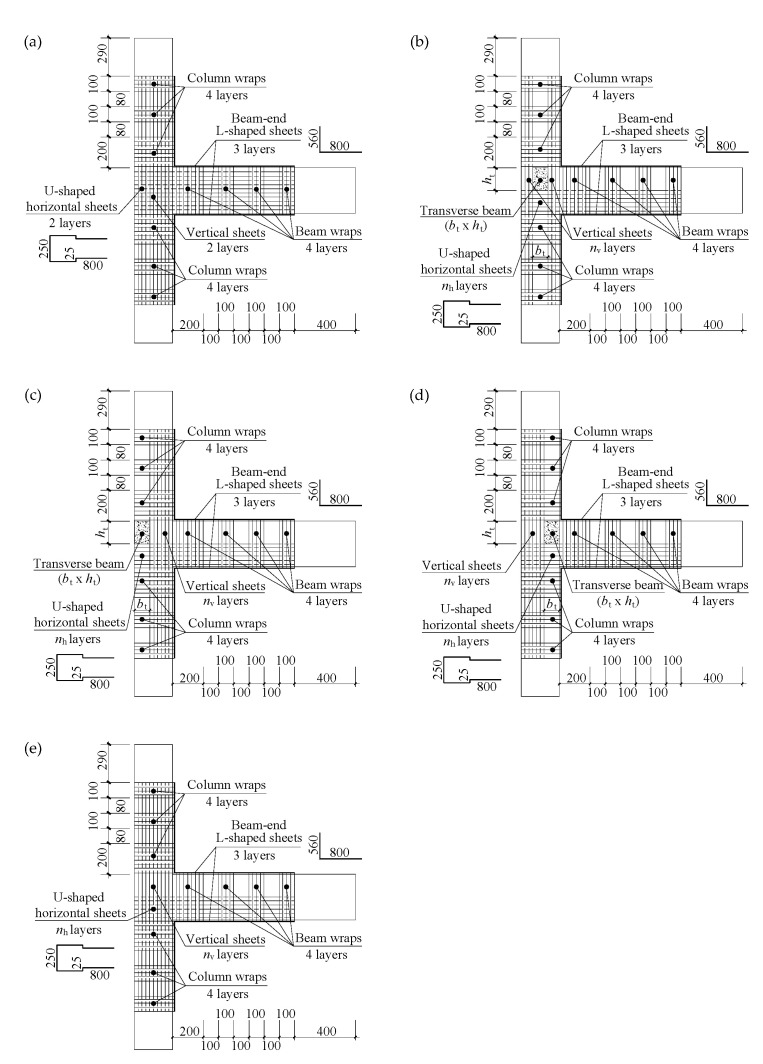
Strengthening schemes (all dimensions are in mm; the values of *b*_t_*, h*_t_, *n*_h_ and *n*_v_ for each of the specimens are listed in Table 1). (**a**) Specimen TJ-3; (**b**) Group DM; (**c**) Group DE; (**d**) Group DI; (**e**) Group SM/SE/SI (the free side of the joint panel).

As shown in Figure 3a, the full surface of the joint panel in Specimen TJ-3 was covered by two layers of U-shaped horizontal CFRP sheets (hereafter referred to as “horizontal sheets”) and two layers of vertical CFRP sheets (hereafter referred to as “vertical sheets”). The horizontal and vertical sheets were used to increase the shear capacity of the joint panel, and were subsequently extended and anchored to the beam end and the column ends (i.e., the innermost ends of the beam and the upper and lower columns that are immediately attached to the joint panel, respectively), respectively. To improve the flexural capacity of the beam end, three layers of L-shaped CFRP sheets were applied to the top and bottom surfaces of the beam end. The width of the L-shaped sheets was equal to that of the beam. The anchorages of the different sheets at the beam end and the column ends were enhanced using four layers of CFRP full wraps. In the practical application of this scheme, the beam wraps and the vertical sheets can be placed through the overlapped holes drilled in the existing slab. Unidirectional carbon fiber sheets with a unit mass of 200 g/m^2^ were used in the current study.

With regard to the shear retrofit of the joints in both Series D and S, the strengthening scheme of Specimen TJ-3 needs to be modified according to the sizes, locations, and numbers of transverse beams, as shown in Figure 3b–e. The differences between the strengthening schemes for the joints in Series D and Specimen TJ-3 were the bond area and the numbers of layers for CFRP sheets. For a joint in Series D, the horizontal sheets were not bonded in the depth range of the transverse beams and no vertical sheets were applied in the width range of the transverse beams. The numbers of CFRP layers in the horizontal and vertical sheets (i.e., *n*_h_ and *n*_v_) varied from 0 to 4. The strengthening scheme for a joint in Series S was identical to that of its counterpart in Series D, except that the vertical sheets were applied to the full surface of the free side of the joint panel, as illustrated in Figure 3e.

A total of 44 FE specimens were designed according to the different combinations of the parameters studied. The specimen labels of the specimens in Series D and S are both ABnXYxy. In this label, AB is the name of a group; n refers to the CFRP amount applied to the joint panel (1, 2, 4, h, v), which is related to values of *n*_h_ and *n*_v_; XY is the width of the transverse beam (*b*_t_) in centimeter; and xy is the overall depth of the transverse beam (*h*_t_) in centimeters. The detailed specimen matrix is presented in Table 1.

### 2.3. Nonlinear FE Modeling

The 3D nonlinear FE models of the specimens were developed in commonly used ANSYS simulation software [38]. The SOLID65 element used to model the nonlinear behavior of concrete is an eight-node solid element with the capability of considering both cracking in tension and crushing in compression. Another eight-node solid element, SOLID45, was used to model the steel plates placed at the two column tips. To model the longitudinal and shear reinforcements, this study used LINK8, a two-node 3D spar element. The plasticity of the steel reinforcements can be simulated by this uniaxial tension–compression element. The externally bonded CFRP sheets were represented by the SHELL41 element, which is defined by four nodes, four thicknesses, a material direction angle, and orthotropic material properties. The element involved membrane stiffness but not bending stiffness.

All the above elements have three degrees of freedom at each node (translation in the nodal x-, y-, and z-directions). A perfect bond was considered between the steel reinforcements and the concrete and between the CFRP sheets and the concrete substrate. Similar conditions were considered in previous numerical investigations on CFRP-strengthened RC joints [30,32]. Especially, all specimens in the current study were designed such that failure would be due to shear in the joint, which ensured that the effect of a series of parameters on the shear capacity of joints can be investigated. As a result, the beam and column bars remained elastic at the ultimate load of each of the specimens. Furthermore, the bond stresses between the concrete and the bars, which were calculated according to the variations of the axial forces along the bars, were checked at different loading steps to ascertain that they fell below the maximum value prescribed in Model Code 2010 [39]. Therefore, a perfect bond between steel reinforcements and concrete was recognized as a rational simplification for FE analysis performed in the current study. Considering that either the horizontal sheets or the vertical sheets had an adequate development length beyond the joint panel and that they were anchored by multiple full wraps, the end debonding failure of the sheets could be avoided. However, the upper limit of CFRP strain in the sheets was assumed to be equal to 0.004, as recommend by the ACI 440.2R-17 [1], for shear strengthening. The value was considered to avoid the loss of aggregate interlock of the concrete. The strain levels in the sheets were also checked at different loading steps to ascertain that they fell below the upper limit.

The key in developing the FE models in both Series D and S is the optimization of the method used for simulating the role of the transverse beams on the CFRP contribution to the joint shear capacity. The philosophy for the optimization can be explained using Equation (1), which is proposed by ACI 440.2R-17 [1], for the seismic strengthening.

*V*_n_ = *V*_n_^*^ + *ψ*_f_*V*_f_ (1)

where *V*_n_ is the shear strength of a strengthened member; *V*_n_^*^ is the shear strength of the origin member; *V*_f_ is the contribution from the FRP system; and *ψ*_f_ is the reduction factor.

The values of *V*_n_^*^ and *V*_f_ of a CFRP-strengthened 3D joint panel are simultaneously influenced by the presence of transverse beams. Nevertheless, a fundamental difference exists between the influences on *V*_n_^*^ and *V*_f_. In the case of a transverse beam with sufficiently large cross-sectional dimensions, e.g., the transverse beam covers at least 3/4 of the face of the joint panel, as prescribed by ACI 318-11 [40], the value of *V*_n_^*^ is increased by the improved concrete contribution from the confinement of the transverse beam. However, this beneficial effect of transverse beams, which is prescribed in detail by existing design codes, was not set as the objective of the current study.

On the other hand, the surface area for bonding the CFRP sheets as well as the continuity of the CFRP confinement for the joint panel was both negatively influenced by the presence of transverse beams. Theoretically, some scenarios of transverse beams may result in a significant decrease in the value of *V*_f_, which was one of the major focuses of the current study. Considering that the geometries of almost all of the CFRP-strengthened joints investigated in the current study were not in accordance with the aforementioned prerequisites for utilizing the beneficial effect of transverse beams [40], the confinement from transverse beams on the crack developments and failure mechanism of the joints is negligible. Therefore, no transverse stub beams were built in the FE models in both Series D and S. The influence of transverse beams on the *V*_f_ value was fully reproduced by restricting the CFRP sheets outside the area of transverse beams at the joint panel. Consequently, the 2D Specimen TJ-1 was used as the control specimen for all of the strengthened specimens to facilitate the comparison of *V*_f_ values related to the different configurations of transverse beams. This simplification can be regarded as a reasonable effort for achieving the objective of the current study.

Based on the convergence study performed with different element sizes, the maximum element size was designated as approximately 50 mm. As a result, most concrete elements were represented by a 50 mm × 50 mm × 50 mm cube. Considering that either the steel elements or the CFRP elements were connected to the concrete elements by sharing the same set of nodes, the lengths of the steel elements and the sizes of the shell elements were consistent with those of the surrounding concrete elements and the substrate concrete elements, respectively.

**Figure 4:**
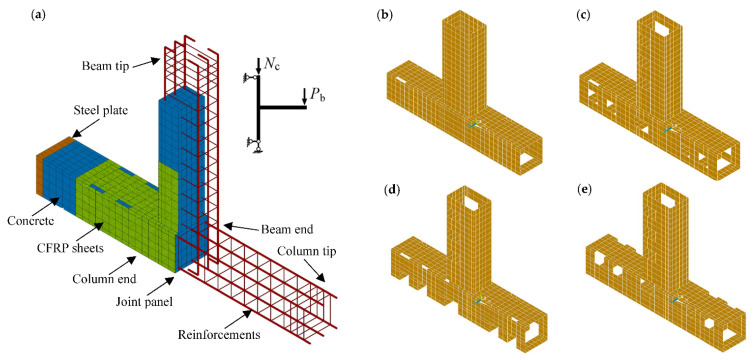
FE models. (**a**) A typical FE model created in ANSYS; (**b**) CFRP system of Specimen TJ-3; (**c**) CFRP system of Specimen DM21015; (**d**) CFRP system of Specimen DE21015; (**e**) CFRP system of Specimen DI21015.

A typical FE model created in ANSYS is shown in Figure 4a. To intuitively reflect the influence of transverse beams on the strengthening schemes, the details of the FE models of the CFRP systems of Specimens TJ-3, DM21015, DE21015 and DI21015 are presented in Figure 4b–e, respectively. A displacement-controlled monotonic load (*P*_b_) was applied at the coupled four lines of the nodes at the top surface of the beam tip, whereas an axial force of 200 kN (*N*_c_) was evenly applied to the nodes on the column upper face (see the schematic illustration of loading in Figure 4a). The column was pinned at both tips by constraining the corresponding degrees of freedom of the nodes at the column centerlines.

### 2.4. Material Properties

The inelastic behavior of the concrete under compressive force was modeled with the Hognestad model [41]. In the current study, the strain associated with the concrete compressive strength and the ultimate strain of concrete were 0.002 and 0.0038, respectively, as recommended by Park and Paulay [42]. Under tension, the behavior of the concrete was assumed to be linear elastic without considering the tensile stress relaxation after cracking [i.e., in SOLID65, KEYOPT(7) = 0]. The Willam-Warnke failure criterion [43] was used for the fracture identification of the concrete. This model uses the concept of a smeared crack model first introduced by Rashid [44], which is especially suitable to the FE analysis of concrete elements. In accordance with the results of the 150 mm^3^ concrete blocks in the experimental tests, the concrete compressive strength was 20.63 MPa. Correspondingly, the concrete tensile strength was taken as 2.04 MPa. The concrete elastic moduli and Poisson’s ratio were assumed to be 30,240 MPa and 0.2, respectively. The value of the shear transfer coefficient for open cracks (*β*_t_) depended on the type of structure, type of load, effect of the dowel action of the reinforcement, and effect of aggregate interlock [45]. Previous researchers [30,31,32,45] selected values of *β*_t_ between 0.125 and 1.0. Considering that the widths of the cracks in the strengthened joints were constrained by the externally bonded CFRP materials that possessed a linear behavior until they reached rupture, a value of 0.5 was used for *β*_t_, i.e., the same value was used in the FE simulation of CFRP-strengthened joints by Alhaddad et al. [30]. Furthermore, the shear transfer coefficient for closed cracks (*β*_c_) was assumed to be 0.95.

An elastic–perfectly plastic model was implemented to simulate the stress–strain behavior of steel materials. In accordance with the steel types used in the experimental tests, the yield strengths of the longitudinal bars and stirrups were 335 and 235 MPa, respectively. The low-strength rebars were widely used in the old-type concrete beam-column joints with deficient shear reinforcement details. The elastic moduli of longitudinal bars, stirrups and steel plates were 200 GPa, 210 GPa and 200 GPa, respectively. The Poisson’s ratio of steel was assumed to be 0.3.

On the basis of the experimental data of carbon fiber and epoxy resin, the elastic properties of CFRP were subsequently predicted using the semi-empirical Chamis model [46]. The Chamis micromechanical model is one of the most used and trusted models for evaluating unidirectional composite materials. As a transversely isotropic material, the elastic properties of the unidirectional CFRP sheets can be defined using five independent engineering constants: longitudinal and transversal Young’s moduli *E*_11_ and *E*_22_, major Poisson’s ratios *ν*_12_ and *ν*_23_, and longitudinal shear moduli *G*_12_. It is noted that direction 1 runs along the fiber. In general, the externally bonded FRP systems can have fiber volumes of 25 to 40% [1]. In the current study, a fiber volume of 40% was assumed. Hence, the thickness of CFRP for each layer was 0.278 mm for the unidirectional carbon fiber sheets with a unit mass of 200 g/m^2^. The additional epoxy resins on the exterior face of CFRP sheets and between two successive CFRP layers were ignored. The five constants of the CFRP sheets calculated according to the Chamis model and the elastic properties of carbon fiber and epoxy resin are listed in Table 2.

The element stiffness matrix for each unidirectional CFRP sheet in the FE models in the current study was determined using the Chamis model, and *E*_22_ was set to a negligible level to improve the convergence behavior of the models. Given that the tensile strength of the epoxy resin is generally much higher than that of the concrete substrate, inter-laminar failure may be rarely observed between the two overlapped unidirectional sheets regardless of whether the fibers in the different sheets are oriented in the same direction. Therefore, to reasonably simplify the modeling, the current study ignored the interaction between two overlapping sheets, but without influencing the overall performance of a CFRP-strengthening system. Consequently, the same set of nodes were shared by the two overlapping sheets whose fibers were oriented in two orthogonal directions. The stiffness matrix of a CFRP element in the overlapping area was assembled by the stiffness matrixes of the sheets.

### 2.5. Experimental Verification

During testing, Specimen TJ-1 had a premature shear failure at the joint panel, whereas Specimen TJ-3 exhibited a more ductile beam-joint failure with the development of a flexural hinge at the beam end.

**Figure 5:**
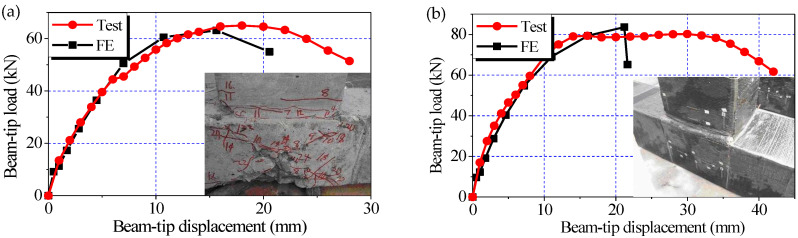
Comparison of the experimental and numerical load-displacement curves. (**a**) Specimen TJ-1; (**b**) Specimen TJ-3.

The comparisons of the experimental and numerical load-displacement curves for Specimens TJ-1 and TJ-3 are shown in Figure 5a,b, respectively. The experimental curves are the envelope ones that are plotted according to the averages of the forward and backward peak loads of the respective first loading cycles at the different beam-tip displacements. Generally, such an envelope curve is almost identical to its counterpart obtained from monotonic loading, considering the fact that the stiffness degradation of a specimen occurring in the following cycles after the first cycle in a displacement-controlled load step has very little influence on the first cycle of the next load step. It is the reason that in this study the envelope curves were used to compare with the numerical curves obtained from monotonic loading. The corresponding failure modes observed in the experimental tests are also presented in the plots.

The comparisons of the beam-tip load versus displacement curves, even to the points of ultimate loading, shows that the results of nonlinear FE and the experimental tests are in good agreement. The differences between the ultimate beam-tip loads obtained from the numerical analysis and their counterparts measured via the experimental tests are negligible. This finding confirms the reliability of the proposed modeling approach when predicting the shear capacities of the original and CFRP-strengthened joints. However, the numerical beam-tip failure displacements were significantly lower than those of the corresponding experimental results for both specimens, which can be attributed to the inability of the current version of ANSYS to model the descending parts of the compressive behavior of concrete [30,31,32]. Nevertheless, the objective of the current study was not influenced by this shortcoming, considering that the softening behavior of the concrete had minimal influence on the contributions of the concrete and CFRP sheets at the loading step in which the joint shear capacity is reached.

## 3. Results

### 3.1. Load Response

**Figure 6:**
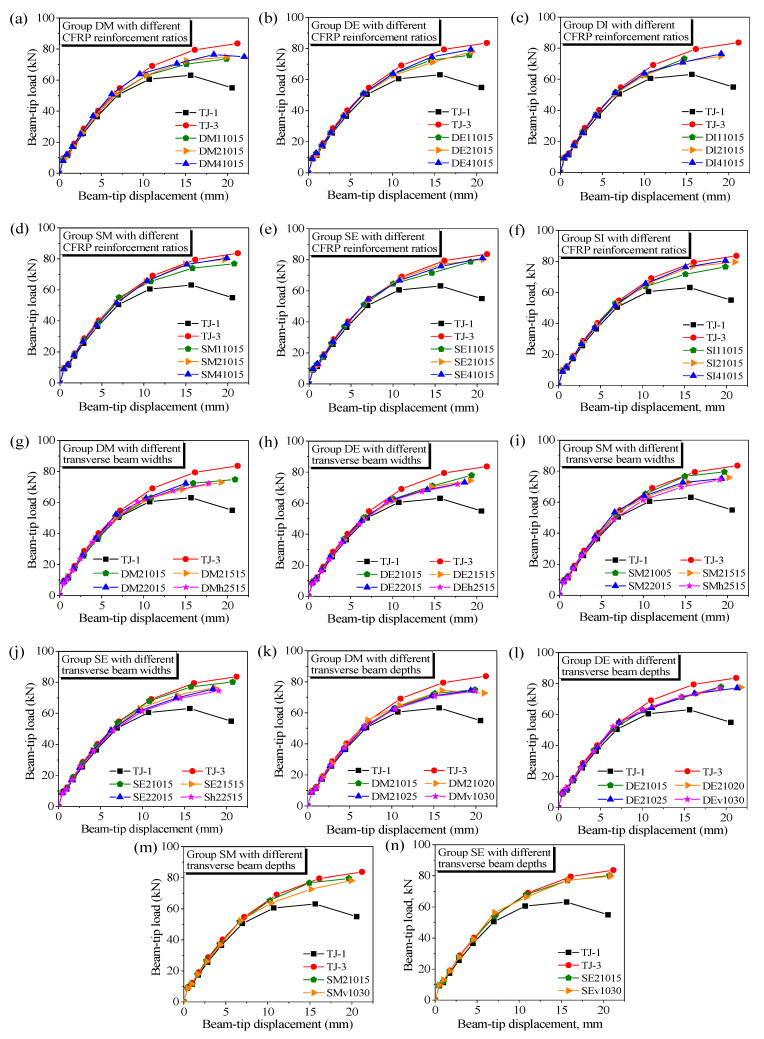
Comparison of the load-displacement curves in different groups. (**a**) Group DM with different CFRP reinforcement ratios; (**b**) Group DE with different CFRP reinforcement ratios; (**c**) Group DI with different CFRP reinforcement ratios; (**d**) Group SM with different CFRP reinforcement ratios; (**e**) Group SE with different CFRP reinforcement ratios; (**f**) Group SI with different CFRP reinforcement ratios; (**g**) Group DM with different transverse beam widths; (**h**) Group DE with different transverse beam widths; (**i**) Group SM with different transverse beam widths; (**j**) Group SE with different transverse beam widths; (**k**) Group DM with different transverse beam depths; (**l**) Group DE with different transverse beam depths; (**m**) Group SM with different transverse beam depths; (**n**) Group SE with different transverse beam depths.

The beam-tip load versus displacement responses were compared for the different groups, as shown in Figure 6a–n. The results of Specimens TJ-1 and TJ-3 are added in each of the plots, and used as the lower and upper bounds of the performance of the CFRP-strengthened 3D joints. The descending parts of the curves are not plotted to provide a tidy illustration.

The FE results of the different joints, expressed in terms of ultimate load, increase in ultimate load, and strengthening efficiency, are summarized in Table 1. The ultimate beam-tip load, which is the maximum beam-tip load obtained during monotonic loading, is used as a simplified representative of the shear capacity of the joint panel, considering the direct link between these two values. The increase in ultimate load is defined as (*F*_i_/*F*_1_) − 1, where *F*_i_ and *F*_1_ are the ultimate beam-tip loads of a given specimen and Specimen TJ-1, respectively. Strengthening efficiency, a new dimensionless index, is introduced to evaluate the residual effect of a CFRP-strengthening system weakened by the presence of transverse beams in comparison with the increase in the joint shear capacity of Specimen TJ-3. The index is defined as (*F*_i_ − *F*_1_)/(*F*_3_ − *F*_1_), where *F*_3_ is the ultimate beam-tip load of Specimen TJ-3.

As shown in Figure 6, the nonlinear portions of the curves of the CFRP-strengthened 3D joints are all lower than that of Specimen TJ-3 to various extents, whereas the initial linear portions of all these curves overlap with one another. An 8.3% to 28.7% increase in the ultimate beam-tip loads of the 3D joints was derived after retrofitting. The corresponding strengthening efficiencies are from 25.3 to 87.5%, as presented in Table 1. The results indicate the negative effect of transverse beams on the CFRP contribution to the shear capacity of the joint panel.

### 3.2. Effect of CFRP Reinforcement Ratio

**Figure 7:**
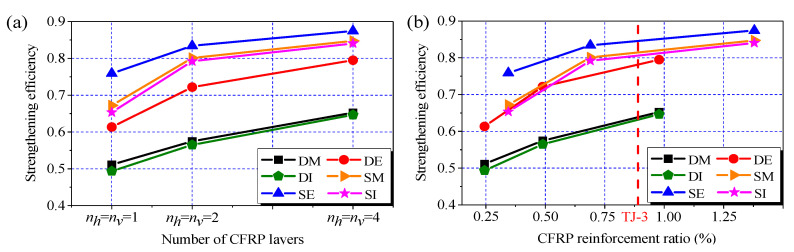
Effect of the CFRP amount applied to the joint panel. (**a**) Strengthening efficiency versus number of CFRP layers; (**b**) Strengthening efficiency versus CFRP reinforcement ratio.

As presented in Figure 6a–f, the ultimate load in each of Groups DE, DM, DI, SE, SM, and SI increased with the number of CFRP layers. The strengthening efficiencies of the joints for the different groups were compared at each level of the CFRP amount applied to the joint panel (i.e., *n*_h_ = *n*_v_ = 1, 2, 4). The curves shown in Figure 7a are illustrated according to the results that correspond to the joints in which the transverse beams have an identical cross section of 100 mm × 150 mm (i.e., width × depth). The curves are further transformed and re-illustrated as a relationship between strengthening efficiency and CFRP reinforcement ratio, as shown in Figure 7b. The CFRP reinforcement ratio of the joint panel (*ρ*_f_) is defined using Equation (2), where *h*_b_ is the overall beam depth, *h*_c_ is the overall column depth, *t*_f_ is CFRP thickness per layer, *n*_t_ is the number of transverse beams, and *b*_c_ is the column width. The CFRP reinforcement ratio of Specimen TJ-3 is also marked in Figure 7b.

*ρ*_f_ = [*n*_h_*t*_f_ (2*h*_b_ − *n*_t_*h*_t_)/*h*_b_ + *n*_v_*t*_f_ (2*h*_c_ − *n*_t_*b*_t_)/*d*_c_]/*b*_c_ (2)

As shown in Figure 7, the strengthening efficiencies for the different groups increased with, but not proportionally to, the number of CFRP layers. The curves exhibit two stages, and most of the increments in the strengthening efficiency are observed at the first stage (i.e., *n*_h_ = *n*_v_ ≤ 2). In the case of *n*_h_ = *n*_v_ = 2, the strengthening efficiencies in Series S were approximately 0.8, whereas those in Series D varied between 0.57 and 0.72. However, when *n*_h_ and *n*_v_ were further increased, the improvements in the strengthening efficiency became limited. Depending on the locations of transverse beams, the overall increase rates of the strengthening efficiency were at the ranges of 15.2–28.6% and 27.6–41.6% in Series D and S, respectively, when *n*_h_ and *n*_v_ both increased from 1 to 4.

An obvious reason for the relatively low efficiencies of the different strengthening schemes for the 3D joints was the loss of bond areas of the CFRP sheets at the joint panels. In the case of *n*_h_ = *n*_v_ = 2, the CFRP reinforcement ratios of the joints in Series D and S were only 55.0% and 78.0% of that of Specimen TJ-3, respectively. By contrast, the strengthening efficiencies in the two series with four layers of CFRP sheets in the two orthogonal directions (i.e., *n*_h_ = *n*_v_ = 4) were still significantly lower than that of Specimen TJ-3. This finding implies that a new shear resistance mechanism, which is less efficient compared with that of Specimen TJ-3, formed in the joint panels of the 3D joints. In particular, the role of externally bonded CFRP materials in confining the concrete cracking in the joint panel was interrupted by the transverse beams. Therefore, increasing the CFRP reinforcement ratio on the joint panel is not feasible to entirely offset the negative effect of the transverse beams on the efficiency of the CFRP system.

### 3.3. Effect of CFRP Layouts

**Figure 8:**
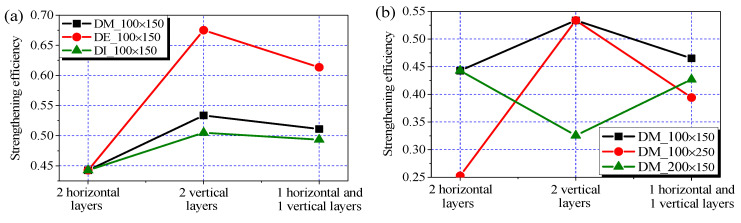
Effect of CFRP layouts. (**a**) Different locations of transverse beam; (**b**) Different cross-section sizes of transverse beam.

The difference in effectiveness between the horizontal and vertical sheets was investigated via three CFRP layouts applied to the joint panels in Series D. The setups included two layers of horizontal sheets (i.e., *n*_h_ = 2, *n*_v_ = 0), two layers of vertical sheets (i.e., *n*_h_ = 0, *n*_v_ = 2), and one layer of CFRP sheet in two orthogonal directions (i.e., *n*_h_ = *n*_v_ = 1). The roles of the locations and cross-section sizes of the transverse beams were compared, as shown in Figure 8a,b, respectively. The curve labels in the figures include information about the locations (DM, DE, and DI) and the cross-section sizes (in millimeter) of the transverse beams. Three kinds of cross sections, namely, 100 mm × 150 mm, 100 mm × 250 mm, and 200 mm × 150 mm, were used. The first cross section was used to render a highly similar CFRP reinforcement ratio between the horizontal and vertical sheets, whereas the two other cross sections were used to represent the cases of the deeper and wider transverse beams, respectively.

As shown in Figure 8a and Table 1, the contributions of the vertical sheets, expressed in terms of strengthening efficiency, were 52%, 21%, and 11% higher than those of the horizontal ones in Groups DE, DM, and DI, respectively. In the case of the deeper transverse beam (i.e., width × depth = 100 mm × 250 mm), the contribution of the vertical sheets was nearly twice that of the horizontal ones, as presented in Figure 8b and Table 1. By contrast, the contribution of the vertical sheets was 26% lower than that of the horizontal ones with the presence of the wider transverse beams (i.e., width × depth = 200 mm × 150 mm). Notably, the overall contribution of the two kinds of sheets in each of the cases was not the sum of the effects of the two kinds of sheets that were used separately. This finding implies that an interaction exists between the strengthening mechanisms of the horizontal and vertical sheets. The interaction can be explained by the coupling effect of the concrete contributions in the horizontal and vertical load paths associated with the horizontal and vertical sheets, respectively. This scenario is similar to that existing between the horizontal and vertical mechanisms in the truss model for shear resistance in the RC joint panel [46].

In conventional RC joints, the effect of column intermediate bars on joint shear capacity is more modest than that of joint hoops [47]. In the case of the CFRP-strengthened 2D joints, a similar conclusion was reached between the horizontal and vertical sheets applied on the joint panels [6]. However, as depicted by the results of the current study, the contribution of the vertical sheets may dominate. A major reason may be that the horizontal sheets were applied beneath the transverse beams only, whereas the vertical sheets were continuously applied in the height ranges of the joint panels.

### 3.4. Effect of Transverse Beam-To-Joint Panel Width Ratio

**Figure 9:**
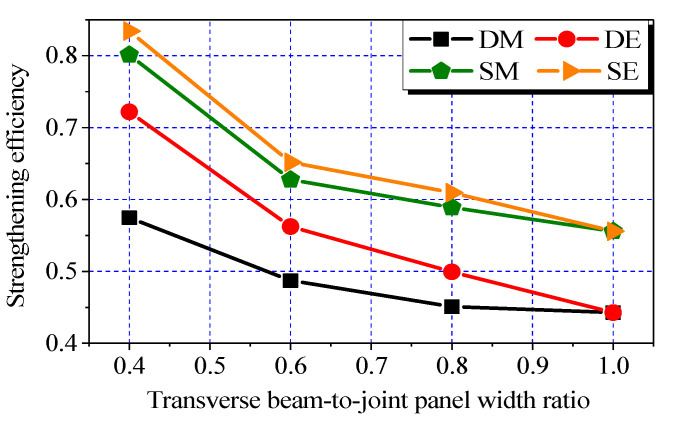
Effect of transverse beam-to-joint panel width ratio.

As shown in Figure 6g–j, the ultimate load in each of Groups DE, DM, SE, and SM decreased with the transverse beam width. The strengthening efficiency versus transverse beam-to-joint panel width ratio (hereafter referred to as “width ratio”) curves of the different groups (DE, DM, SE, and SM) are presented in Figure 9. The width ratio is defined as *b*_t_/*h*_c_. In each of the groups, the width of transverse beams was increased from 100 to 250 mm at an increment of 50 mm, while the overall depth of transverse beams remained unchanged at 150 mm. Two layers of CFRP sheets were applied to each side of the joint panels in the two orthogonal directions (i.e., *n*_h_ = *n*_v_ = 2) except for those with a width ratio of 1.0 (i.e., *n*_h_ = 2, *n*_v_ = 0).

Except for those in Group DM, the strengthening efficiencies ranged from 0.72 to 0.83 for the joints with width ratios of 0.4. This finding suggests the limited negative effect of the narrow transverse beams on the CFRP systems. However, significant decreases in the strengthening efficiencies of the different groups were also observed when the width ratio was further increased. When the width ratio was increased from 0.4 to 1.0, the overall decrease rates of the strengthening efficiencies of the joints in Groups DM, DE, SM, and SE were 23%, 39%, 31%, and 33%, respectively. At the width ratio of 0.6, the strengthening efficiency in Group DM was lower than 0.5, whereas those in the other groups decreased to approximately 0.6. Hence, the width ratio of 0.6 can be used as a basis to evaluate the feasibility of a retrofit case in practice.

The observed significant influence of width ratio can be explained by the proportional decrease in the bond areas of the vertical sheets in relation to the increases in width of transverse beams. As discussed in the previous section, the contributions of the vertical sheets were higher than those of the horizontal ones in most cases.

### 3.5. Effect of Transverse Beam-To-Joint Panel Height Ratio

As illustrated in Figure 6k–n, the ultimate load in each of Groups DE, DM, SE, and SM decreased with the transverse beam height. The strengthening efficiency versus transverse beam-to-joint panel height ratio (hereafter referred to as “height ratio”) curves of the different groups (DE, DM, SE, and SM) are shown in Figure 9. The depths of transverse beams of 150, 200, 250, and 300 mm were selected, whereas the width of the transverse beams remained unchanged at 100 mm. The height ratio is defined as *h*_t_/*h*_b_. Two layers of CFRP sheets were applied to each side of the joint panels in the two orthogonal directions (i.e., *n*_h_ = *n*_v_ = 2) except for those with a height ratio of 1.0 (i.e., *n*_h_ = 0, *n*_v_ = 2).

**Figure 10:**
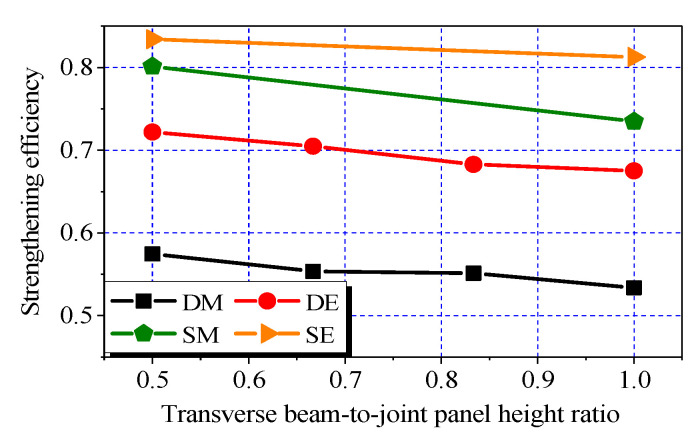
Effect of transverse beam-to-joint panel height ratio.

As shown in Figure 10, the decrements in the curves were negligible when the height ratio was increased from 0.5 to 1.0. This finding indicates that the horizontal sheets were not effective when their bond areas were limited to the bottom halves of the joint panels. Hence, the height ratio exhibited a minor role on the effectiveness of the CFRP systems in comparison with the width ratio. Accordingly, the relatively higher strengthening efficiencies (i.e., 0.81, 0.74, 0.68, and 0.53 for the joints in Groups SE, SM, DE, and DM, respectively) were achieved at the height ratio of 1.0.

### 3.6. Effect of Transverse Beam Location

As shown in Figure 7, Figure 8, Figure 9 and Figure 10, the strengthening efficiency increased considerably when the location of transverse beams was moved away from the inner edge of the joint panel. The joints in Group DE had the strengthening efficiencies of up to 134% in relation to their counterparts with an identical CFRP reinforcement ratio in Group DI, as shown in Figure 7 and Table 1. Figure 7 also shows that Curve DM is extremely close to that of Curve DI, which implies that the beneficial role of the location of transverse beams on the CFRP became negligible when the transverse beams were at the inner halves of the joint panels. Moreover, as shown in Figure 9, the difference between Curves DE and DM is decreased to 0 when width ratio is increased to 1.0, which confirms that only the contribution of the vertical sheets was influenced by the location of transverse beams.

**Figure 11:**
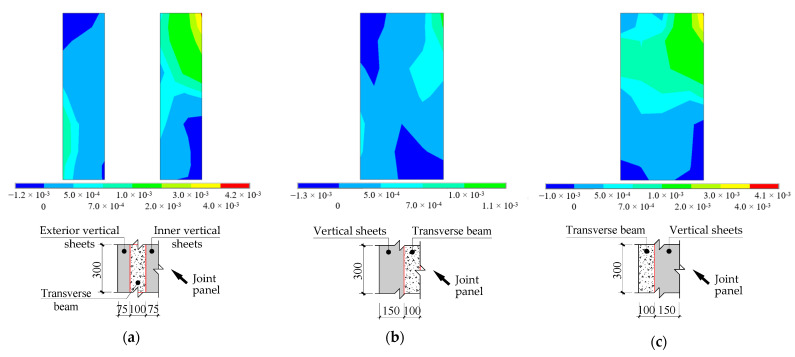
Distributions of longitudinal strains in vertical CFRP sheets (positive and negative values indicate tensile and compressive strain, respectively). (**a**) Specimen DMv1030; (**b**) Specimen DIv1030; (**c**) Specimen DEv10.

**Figure 12:**
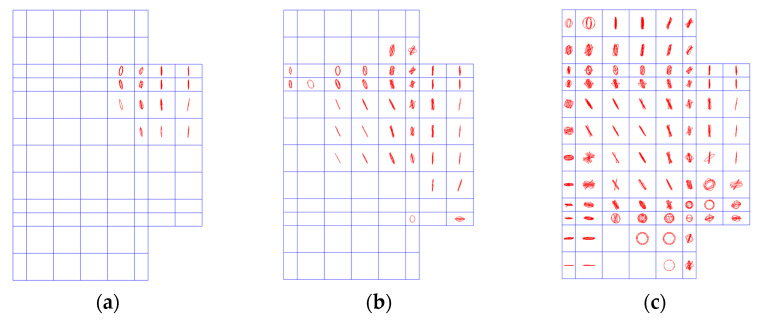
Crack propagation in Specimen TJ-1. (**a**) 17.9% of ultimate load; (**b**) 40.6% of ultimate load; (**c**) 95.9% of ultimate load.

To understand the reason behind the effect of the location of transverse beams, the distributions of strains along the fiber direction in the vertical sheets at the joint panels of Specimens DIv1030, DMv1030, and DEv1030 were compared in Figure 11. To clarify the location and area of the vertical CFRP sheets at the joint panel, a schematic illustration is added to each of Figure 11a–c. The strain distributions were obtained under the respective ultimate beam-tip loads of the specimens. To further explain the strain distributions, the history of the crack propagation recorded at the different loading steps of Specimen TJ-1 is presented in Figure 12. At the centroid of an element, cracking is shown with a circle outline in the plane of the crack, and crushing is shown with an octahedron outline.

As shown in Figure 11, the overall strain level in the vertical sheets is increased when the transverse beams are relocated from the inner edge to the exterior edge of the joint panel. As presented in Figure 11a, the vertical sheets were divided into two halves (i.e., inner and exterior parts), and the maximum strain (approximately 4000 με) in the inner part was nearly four times that of the exterior part. The finding implies that at least half of the vertical sheets were not sufficiently utilized. In the case of the inner edge alignment (see Figure 11b), the overall strain level in the vertical sheets are all less than 1000 με (similar to the exterior part in Figure 11a). On the other hand, a more uniform strain distribution with a relatively higher strain level (i.e., the maximum strain is approximately equal to 4000 με) was observed at the upper part of the vertical sheets when the transverse beams were aligned with the exterior edge of the joint panel (see Figure 11c). However, the strain levels at the lower part of the vertical sheets were all less than 500 με in all cases. As shown in Figure 12, the shear cracks of the joint panel initially appeared at the upper corner adjacent to the beam end and extended diagonally downward. This finding confirms that the regions of the vertical sheets near the beam realized the better confinement on the crack propagation.

### 3.7. Effect of the Number of Transverse Beams

Figure 7, Figure 9 and Figure 10 show that the CFRP-strengthening systems for Series S achieved better effects in comparison with their counterparts for Series D. In Figure 7a and Table 1, the joints in Series S have the strengthening efficiencies as high as approximately 140% in relation to the corresponding joints in Series D. In Figure 7b, the strengthening efficiencies of the joints in Series S are more than 0.8 when the CFRP reinforcement ratio is set to at least 0.007. In Figure 9 and Figure 10, the strengthening efficiencies in Series S are beyond 0.8 when the width ratio and the height ratio are no more than 0.4 and 0.5, respectively. By contrast, no such ranges of the high strengthening efficiency are observed in the corresponding curves of Series D in the plots.

The significant strengthening effect of the joints in Series S was primarily due to the contribution of the CFRP sheets that were continuously applied on the free sides of the joint panels. The result suggests the existence of not only a relatively higher CFRP reinforcement ratio, but also the formation of a more efficient shear mechanism, in the joint panels.

## 4. Discussion

**Figure 13:**
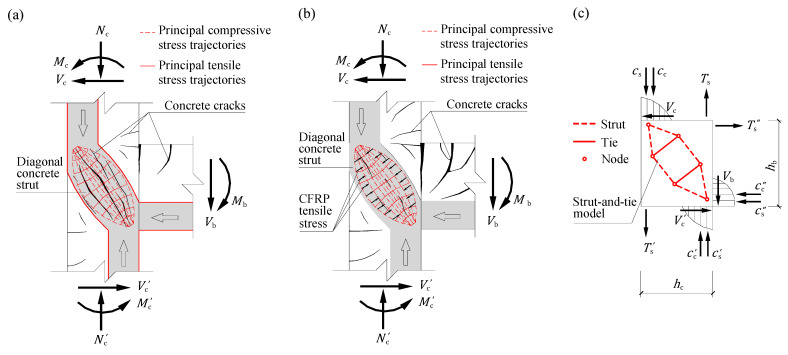
Shear mechanism of CFRP-retrofitted exterior beam-column joints. (**a**) Classical compression strut mechanism formed in lightly reinforced concrete beam-column joints; (**b**) Role of externally bonded CFRP sheets on enhancing shear behavior of joint panel; (**c**) Proposed strut-and-tie model.

Based on the analysis of the experimental and numerical results obtained in this study, the shear mechanism of the CFRP-retrofitted exterior beam-column joints is explained in Figure 13 and discussed qualitatively in this section.

The shear mechanism for the lightly reinforced concrete exterior beam-column joints can be explained by the classical compression strut mechanism [48] formed in the joint panels, as illustrated in Figure 13a. In the model, the axial loads, shear forces and bending moments acting on the upper and lower column ends (i.e., *N*_c_, *V*_c_, *M*_c_, *N*_c_′, *V*_c_′ and *M*_c_′) and the beam end (i.e., *V*_b_ and *M*_b_) are transmitted across the joint panel via the diagonal concrete strut. As shown by the shaded region of the joint panel outlined by the red curves in the plot, the diagonal concrete strut is bottle-shaped, i.e., the cross section of the end is significantly smaller than that of the middle. The diagonal concrete strut represents the flow of concentrated compressive stresses in the concrete, as revealed by the principal stress trajectories simplified from the simulation results of Specimen TJ-1. With the increase of the shear action for the joint panel, short and thin cracks parallel to the diagonal concrete strut axis will occur and further develop and connect with each other to form several major cracks that will eventually lead to the collapse of the diagonal concrete strut. This shear failure process of the lightly reinforced concrete joint panels has been verified via the simulation results shown in Figure 12.

The role of the externally bonded CFRP sheets in enhancing the shear capacity of the lightly reinforced concrete joint panel is illustrated in Figure 13b. As a retrofit configuration with a passive nature, the CFRP sheets bonded to the joint panel begin to be tensioned after the micro-cracks appear in the concrete and have a contribution in confining the further development of the micro-cracks (i.e., the lateral expansion and degradation of the diagonal concrete strut). Therefore, a new shear mechanism that consists of the diagonal concrete strut and the externally bonded CFRP sheets forms in the joint panel. As a result, the mode of failure for the CFRP-retrofitted joint could be transferred from the shear failure of the joint panel to the flexible one at the beam end, which means that a much more ductile behavior will be achieved. However, the continuous confinement provided by the CFRP sheets along the diagonal concrete strut axis only exists in the case of the one-way joints and the 3D ones in Series S, because at least one side of the joint panel can be fully covered by CFRP sheets. For those in Series D, the presence of the two transverse beams will result in a discontinuity in the confinement obtained from the CFRP sheets. This is the reason why the strengthening efficiencies of the joints in Series D are always significantly lower than those of their counterparts in Series S as presented in Section 3.7.

Although the CFRP sheets are used to compensate the lack of stirrups in the joint panel, the classical truss mechanism [48] generally used for the shear design of RC beam-column joints is no longer applies to the CFRP-retrofitted ones. The reason is that the truss mechanism is based on the basic prerequisite of the yield of shear reinforcements, whereas CFRP sheets are a purely elastic-brittle material. In such cases, the approach of the strut-and-tie models [49] is more suitable. The strut-and-tie models are most appropriately used for the design of the disturbed regions that are characterized by a complex flow of internal stresses. The proposed strut-and-tie model used to describe the shear mechanism of the CFRP-retrofitted joint panels and evaluate the respective contributions of concrete and CFRP sheets is illustrated in Figure 13c. In the model, the bottle-shaped diagonal concrete strut is replaced by a group of compressive struts, whereas the contribution of the CFRP sheets is simplified to the two tension ties perpendicular to the two mutually parallel struts. The adjacent concrete struts and CFRP ties are interconnected at the nodal zones to form a structural system. To further clarify the boundary conditions of the proposed model, the force acting on the column-end and beam-end cross sections are marked in the plot, where *C*_c_, *C*_c_′, and *C*_c_″ are the upper column-end, lower column-end and beam-end concrete compression forces, respectively; *C*_s_, *C*_s_′, and *C*_s_″ are the corresponding longitudinal reinforcement compression forces, respectively; *T*_s_, *T*_s_′, and *T*_s_″ are the corresponding longitudinal reinforcement tension forces, respectively.

Under reasonable assumptions, the shear capacities of the one-way CFRP-retrofitted joint panels can be predicted by the proposed model according to the general approach for strut-and-tie models [40,49]. For instance, the following simple assumptions could be used: (1) the thickness of the concrete struts is taken as the column width *h*_b_; (2) the total width of the two mutually parallel concrete struts is taken as (*h*_c_^2^ + *h*_b_^2^)^1/2^/2; (3) the total width of CFRP ties is taken as (*h*_c_^2^ + *h*_b_^2^)^1/2^/2; (4) the respective stresses in the horizontal and vertical CFRP sheets are calculated according to the method used for the conventional CFRP laminates with various ply angles in different layers; (5) the CFRP effective strain could be calibrated by the average tensile strain of the CFRP sheets over the total width of the CFRP ties, or determined by referring to the simple formulae proposed by ACI 440.2R-17 [1] for the bonded face plies.

The above method would also be applied to the simplified design of the CFRP-retrofitted joint panel in Series S, if it is conservatively assumed that the two CFRP ties consist of only the vertical CFRP sheets applied to the full surface of the free side of the joint panel. When the proposed model is used for Series D, however, the respective widths of the two CFRP ties must be determined according to the size and location of the transverse beams. In this case, the ultimate load capacity of the proposed model depends on the weaker CFRP tie. Furthermore, each of the CFRP ties should be checked for its potential to span the major concrete cracks, when CFRP sheets are discretely bonded to the joint panel due to the presence of the transverse beams. Correspondingly, the CFRP effective strains might vary with the ties. Obviously, the method for Series D can be used to further improve the calculation accuracy of the proposed model in the case of Series S. The specific procedure is to add the role of the CFRP sheets associated with the side with a transverse beam to the tensile property of the ties formed by the vertical CFRP sheets at the free side. Therefore, to develop the analytical relationships between the geometric characteristics of the discrete CFRP sheets and the capacity of the CFRP ties is the focus of the next phase of this study. The objective could be achieved using a semi-empirical approach based on the compatibility conditions for joint panels.

Moreover, 3D interior beam-column joints possess the boundary conditions and reinforcement details that differ substantially from those of exterior joints. Especially, plastic hinges are allowed to be developed at the column ends of the interior joints, when the gravity-load-designed RC frame structures are upgraded to resist the large lateral seismic forces according to a limited ductility demand. As a result, the roles of the parameters investigated in this study need to be quantified again in the case of the 3D interior joints.

## 5. Conclusions

This paper presents a detailed process for the nonlinear FE simulation of CFRP-strengthened 3D RC joints. The accuracy of the approach to predict the shear capacity of the joint panel was verified against experimental observations. The findings increased the confidence of using the present FE model to quantify the influence of a series of parameters related to the presence of transverse beams on the effectiveness and efficiency of the externally bonded CFRP systems. The main conclusions can be summarized as follows:

- The strengthening efficiencies of the 3D joints derived in this study were from 25.3% to 87.5%. The results confirmed the effectiveness of the CFRP strengthening technique with regard to the relatively wide ranges of the parameters studied, while the negative effect from transverse beams on the CFRP contribution was observed.
- Considering that the continuity of the CFRP confinement for a joint panel was interrupted by transverse beams, increasing the CFRP reinforcement ratio of the joint panel cannot entirely offset the negative effect from transverse beams on the efficiency of the CFRP system.
- With regard to the relatively wide ranges of the parameters studied, the strengthening efficiencies of the joints with a single transverse beam were at least 0.8.
- The strengthening efficiencies of the joints each with two transverse beams were much lower than their counterparts each with a single transverse beam, and were no more than 0.8 in the studied cases.
- Unlike the conclusion drawn from the results of 2D joints, the strengthening effect of a joint with two transverse beams may be dominated by the contribution of the vertical sheets.
- Significant decreases in the strengthening efficiencies of the 3D joints were observed when the width ratio was increased. A width ratio of 0.6 can be used as a basis to evaluate the feasibility of retrofitting.
- In comparison with the width ratio, the height ratio exhibited a minor role in the strengthening efficiencies of the CFRP systems. Increasing the height ratio beyond 0.5 did not further increase the negative effect from transverse beams on the CFRP contribution.
- The strengthening efficiency of the vertical sheets increased considerably when the location of the transverse beams moved away from the inner edge of the joint panel. This result was confirmed by the strain distributions in the vertical sheets and the course of crack propagation in the joint panel.
- A strut-and-tie model is proposed for describing the shear mechanism of the CFRP-retrofitted joint panel and evaluating the respective contributions of the concrete and CFRP sheets in the shear mechanism. The application of the model is qualitatively discussed in accordance with the different types of CFRP-retrofitted joint panels.

## Figures and Tables

**Table 1:** Specimen details and nonlinear FE analysis results.

		Transverse Beam		CFRP Layouts at the Joint Panel	Numerical Results		
Series	Specimen ID	*b*_t_ (mm)	*h*_t_ (mm)		Ultimate Load (kN)	Increase ^1^ (%)	Efficiency ^2^ (%)
R	TJ-1	-	-	-	63.1	-	-
	TJ-3	-	-	*n*_h_ = *n*_v_ = 2	83.6	32.7	100
D	DM11015, DE11015, DI11015	100	150	*n*_h_ = *n*_v_ = 1	73.6, 75.7, 73.2	16.7, 20.0, 16.2	51.1, 61.4, 49.4
	DM11025	100	250		71.1	12.9	39.4
	DM12015	200	150		71.8	13.9	42.7
	DM21015, DE21015, DI21015	100	150	*n*_h_ = *n*_v_ = 2	74.9, 77.9, 74.7	18.8, 23.6, 18.5	57.5, 72.2, 56.5
	DM21020, DE21020	100	200		74.4, 77.5	18.1, 23.0	55.4, 70.5
	DM21025, DE21025	100	250		74.4, 77.1	18.0, 22.3	55.1, 68.3
	DM21515, DE21515	150	150		73.1, 74.6	15.9, 18.4	48.7, 56.2
	DM22015, DE22015	200	150		72.3, 73.3	14.7, 16.3	45.1, 49.9
	DM41015, DE41015, DI41015	100	150	*n*_h_ = *n*_v_ = 4	76.5, 79.4, 76.4	21.3, 26.0, 21.2	58.5, 71.3, 58.2
	DMh2515	250	150	*n*_h_ = 2, *n*_v_ = 0	72.1	14.5	44.3
	DMh2525	250	250		68.2	8.3	25.3
	DMv1030, DEv1030, DIv1030	100	300	*n*_h_ = 0, *n*_v_ = 2	74.0, 76.9, 73.4	17.4, 22.1, 16.5	53.3, 67.5, 50.5
	DMv2030	200	300		69.7	10.6	32.6
S	SM11015, SE11015, SI11015	100	150	*n*_h_ = *n*_v_ = 1	76.9, 78.7, 74.9	22.0, 24.8, 18.8	67.2, 75.9, 57.7
	SM21015, SE21015, SI21015	100	150	*n*_h_ = *n*_v_ = 2	79.5, 80.2, 76.8	26.2, 27.3, 21.8	80.1, 83.4, 66.7
	SM21515, SE21515	150	150		76.0, 76.5	20.5, 21.3	62.8, 65.2
	SM22015, SE22015	200	150		75.2, 75.6	19.2, 19.9	58.9, 60.1,
	SM41015, SE41015, SI41015	100	150	*n*_h_ = *n*_v_ = 4	80.5, 81.0, 77.6	27.7, 28.7, 23.2	84.8, 87.5, 70.9
	SMh2515	250	150	*n*_h_ = 2, *n*_v_ = 0	74.5	18.2	55.6
	SMv1030, SEv1030, SIv1030	100	300	*n*_h_ = 0, *n*_v_ = 2	78.2, 79.8, 76.0	24.0, 26.5, 20.6	73.5, 81.3, 63.2

^1^ Increase = percent difference with Specimen TJ-1. ^2^ Efficiency = strengthening efficiency.

**Table 2:** Summary of elastic properties of carbon fiber, epoxy resin and CFRP.

Property	Carbon Fiber ^1^	Epoxy Resin ^1^	CFRP ^2^
*E*_11_ (MPa)	232,000	3450	94,870
*E*_22_ (MPa)	15,000	3450	6725
*G*_12_ (MPa)	24,000	1280	3190
*v*_12_	0.279	0.35	0.32
*v*_23_	0.49	0.35	0.39

^1^ Experimental data were taken from Younes et al. [46]. ^2^ Predicted by the Chamis model for the wet layup system with a fiber volume of 40%.

## Data Availability

Data is contained within the article.

## References

[1] American Concrete Institute (ACI) (2017). Guide for the Design and Construction of Externally Bonded FRP Systems for Strengthening Concrete Structures.

[2] Teng J.G., Chen J.F., Smith S.T., Lam L. (2002). FRP Strengthened RC Structures.

[3] Wang B., Wu X., Sun Z., Liang J., Mao X., Bi T., Song Y., Yang W. (2021). Experimental investigation on low-velocity impact behavior of CFRP wraps in presence of concrete substrate. Constr. Build. Mater..

[4] Gergely I., Pantelides C.P., Nuismer R.J., Reaveley L.D. (1998). Bridge pier retrofit using fiber-reinforced plastic composites. J. Compos. Constr..

[5] Gergely J., Pantelides C.P., Reaveley L.D. (2000). Shear strengthening of RCT-joints using CFRP composites. J. Compos. Constr..

[6] Antonopoulos C., Triantafillou T.C. (2003). Experimental investigation of FRP-strengthened RC beam-column joints. J. Compos. Constr..

[7] Ghobarah A., El-Amoury T. (2005). Seismic rehabilitation of deficient exterior concrete frame joints. J. Compos. Constr..

[8] Pantelides C.P., Okahashi Y., Reaveley L.D. (2008). Seismic rehabilitation of reinforced concrete frame interior beam-column joints with FRP composites. J. Compos. Constr..

[9] Tsonos A.G. (2008). Effectiveness of CFRP-jackets and RC-jackets in post-earthquake and pre-earthquake retrofitting of beam–column sub-assemblages. Eng. Struct..

[10] Akguzel U., Pampanin S. (2010). Effects of variation of axial load and bidirectional loading on seismic performance of GFRP retrofitted reinforced concrete exterior beam-column joints. J. Compos. Constr..

[11] Li B., Kai Q., Xue W. (2012). Effects of eccentricity on the seismic rehabilitation performance of nonseismically detailed interior beamwide column joints. J. Compos. Constr..

[12] Garcia R., Jemaa Y., Helal Y., Guadagnini M., Pilakoutas K. (2014). Seismic strengthening of severely damaged beam-column RC joints using CFRP. J. Compos. Constr..

[13] Realfonzo R., Napoli A., Pinilla J.G.R. (2014). Cyclic behavior of beam-column joints strengthened with FRP systems. Constr. Build. Mater..

[14] Singh V., Bansal P.P., Kumar M., Kaushik S.K. (2014). Experimental studies on strength and ductility of CFRP jacketed reinforced concrete beam-column joints. Constr. Build. Mater..

[15] Del Vecchio C., Di Ludovico M., Balsamo A., Prota A., Manfredi G., Dolce M. (2014). Experimental investigation of exterior RC beam-column joints retrofitted with FRP systems. J. Compos. Constr..

[16] Hadi M.N.S., Tran T.M. (2016). Seismic rehabilitation of reinforced concrete beam-column joints by bonding with concrete covers and wrapping with FRP composites. Mater. Struct..

[17] Mostofinejad D., Akhlaghi A. (2016). Experimental investigation of the efficacy of EBROG method in seismic rehabilitation of deficient reinforced concrete beam-column joints using CFRP sheets. J. Compos. Constr..

[18] Mostofinejad D., Hajrasouliha M. (2018). Shear retrofitting of corner 3D-reinforced concrete beam-column joints using externally bonded CFRP reinforcement on grooves. J. Compos. Constr..

[19] Pohoryles D.A., Melo J., Rossetto T., D’Ayala D., Varum H. (2018). Experimental comparison of novel CFRP retrofit schemes for realistic full-scale RC beam-column joints. J. Compos. Constr..

[20] Faleschini F., Gonzalez-Libreros J., Zanini M.A., Hofer L., Sneed L., Pellegrino C. (2019). Repair of severely-damaged RC exterior beam- column joints with FRP and FRCM composites. Compos. Struct..

[21] Allam K., Mosallam A.S., Salama M.A. (2019). Experimental evaluation of seismic performance of interior RC beam-column joints strengthened with FRP composites. Eng. Struct..

[22] Ma C., Wang Z., Smith S.T. (2020). Seismic performance of large-scale RC eccentric corner beam-column-slab joints strengthened with CFRP systems. J. Compos. Constr..

[23] Kothapalli N.K., Chidambaram R.S., Agarwal P. (2022). Cyclic evaluation of severely damaged RC frames repaired and strengthened through FRP-wrapped coupler-box confinement. J. Compos. Constr..

[24] Antonopoulos C., Triantafillou T.C. (2002). Analysis of FRP-strengthened beam-column joints. J. Compos. Constr..

[25] Bousselham A. (2010). State of research on seismic retrofit of RC beam-column joints with externally bonded FRP. J. Compos. Constr..

[26] Akguzel U., Pampanin S. (2012). Assessment and design procedure for the seismic retrofit of reinforced concrete beam-column joints using FRP composite materials. J. Compos. Constr..

[27] Del Vecchio C., Di Ludovico M., Prota A., Manfredi G. (2015). Analytical model and design approach for FRP strengthening of non-conforming RC corner beam–column joints. Eng. Struct..

[28] Bossio A., Fabbrocino F., Lignola G.P., Prota A., Manfredi G. (2015). Simplified model for strengthening design of beam-column internal joints in reinforced concrete frames. Polymers.

[29] Bossio A., Fabbrocino F., Lignola G.P., Prota A., Manfredi G. (2017). Design oriented model for the assessment of T-shaped beam-column joints in reinforced concrete frames. Buildings.

[30] Alhaddad M.S., Siddiqui N.A., Abadel A.A., Alsayed S.H., Al-Salloum Y.A. (2012). Numerical investigations on the seismic behavior of FRP and TRM upgraded RC exterior beam-column joints. J. Compos. Constr..

[31] Dalalbashi A., Eslami A., Ronagh H.R. (2013). Numerical investigation on the hysteretic behavior of RC joints retrofitted with different CFRP configurations. J. Compos. Constr..

[32] Eslami A., Ronagh H.R. (2016). Numerical investigation on the seismic retrofitting of RC beam-column connections using flange-bonded CFRP composites. J. Compos. Constr..

[33] Mosallam A., Allam K., Salama M. (2019). Analytical and numerical modeling of RC beam-column joints retrofitted with FRP laminates and hybrid composite connectors. Compos. Struct..

[34] Pohoryles D.A., Melo J., Rossetto T., Varum H., Bisby L. (2019). Seismic retrofit schemes with FRP for deficient RC beam-column joints: State-of-the-art review. J. Compos. Constr..

[35] Wang B., Zhu H., Wu X., Zhang N., Yan B. (2020). Numerical investigation on low-velocity impact response of CFRP wraps in presence of concrete substrate. Compos. Constr..

[36] Titirla M.D., Chalot A., Michel L., Ferrier E. (2020). 3D Finite element modelling of novel strengthening solutions for RC wall/slab connections. Ing. Sismica-Ital..

[37] Earij A., Alfano G., Cashell K., Zhou X. (2017). Nonlinear three–dimensional finite–element modelling of reinforced–concrete beams: Computational challenges and experimental validation. Eng. Fail. Anal..

[38] ANSYS Inc. (2012). ANSYS (version 14.5). Windows.

[39] The International Federation for Structural Concrete (2010). Fib Model Code for Concrete Structures.

[40] American Concrete Institute (ACI) (2011). Building Code Requirements for Structural Concrete and Commentary.

[41] Hognestad E., Hanson N.W., McHenry D. (1955). Concrete stress distribution in ultimate strength design. ACI J. Proc..

[42] Park R., Paulay T. (1975). Reinforced Concrete Structures.

[43] William K.J., Warnke E.P. Constitutive model for the triaxial behavior of concrete. Proceedings of the International Association for Bridge and Structural Engineering (IABSE) Seminar on Concrete Structures Subjected to Triaxial Stress.

[44] Rashid Y.R. (1968). Ultimate strength analysis of prestressed concrete pressure vessels. Nucl. Eng. Des..

[45] Kwak H.G., Fillippou F.C. (1990). Finite Element Analysis of Reinforced Concrete Structures under Monotonic Loads.

[46] Younes R., Hallal A., Fardoun F., Chehade F.H., Hu N. (2012). Comparative review study on elastic properties modeling for unidirectional composite materials. Composites and Their Properties.

[47] Kassem W. (2016). Strut-and-tie modelling for the analysis and design of RC beam-column joints. Mater. Struct..

[48] Paulay T., Priestley M.J.N. (1992). Seismic Design of Reinforced Concrete and Masonry Buildings.

[49] American Concrete Institute (ACI) (1999). Recent Approaches to Shear Design of Structural Concrete.

